# *Ae1/Sbe1* maize-derived high amylose improves gut barrier function and ameliorates type II diabetes in high-fat diet-fed mice by increasing *Akkermansia*

**DOI:** 10.3389/fnut.2022.999020

**Published:** 2022-09-29

**Authors:** Weiwei Qi, Jingchao Liu, Tante Yu, Shengchan Huang, Rentao Song, Zhenyi Qiao

**Affiliations:** ^1^Shanghai Key Laboratory of Bio-Energy Crops, School of Life Sciences, Shanghai University, Shanghai, China; ^2^State Key Laboratory of Dairy Biotechnology, Shanghai Engineering Research Center of Dairy Biotechnology, Dairy Research Institute, Bright Dairy & Food Co., Ltd., Shanghai, China; ^3^State Key Laboratory of Plant Physiology and Biochemistry, National Maize Improvement Center, College of Agronomy and Biotechnology, China Agricultural University, Beijing, China

**Keywords:** *ae1/sbe1* maize, amylose, type II diabetes, *Akkermansia*, gut barrier

## Abstract

Type II diabetes mellitus (T2DM) has its origins in chronic inflammation due to immune dysregulation. Improving chronic inflammation can significantly reduce the probability of T2DM and the rate of disease progression. Resistance to starch 2 (RSII) high-amylose maize starch (HAMS) has been widely implicated in the improvement and regulation of T2DM. However, its exact molecular mechanisms have not been fully discovered. Here, we used CRISPR/Cas9 technology to knock out two starch-branching enzyme genes, *Ae1* and *Sbe1*, in maize to obtain mutants containing higher levels of HAMS. In experiments in which HAMS was fed to mice on a high-fat diet (HFD), we confirmed the function of HAMS in ameliorating hyperglycemia. Mechanistically, we found that HAMS improves the gut barrier function by increasing the *Akkermansia* abundance in the gut. This increase led to the alleviation of chronic inflammation in mice on a HFD, resulting in improved insulin sensitivity and a decrease in blood glucose.

## Introduction

Advances in life sciences and medicine have led to a deeper understanding of health. Many diseases that were once thought to be unrelated are intrinsically linked. For example, research has confirmed that although the direct cause of type II diabetes is increased insulin resistance, the underlying cause is a chronic inflammatory response (1–6). Changes in immune function not only contribute to the development of type II diabetes but are also involved in the progression of many other diseases, such as Alzheimer's disease, malignancies, and other chronic diseases (7, 8). Inflammation is a major driver in the progression of many diseases (especially in old age) (9–11). Conversely, improving immune function can greatly alleviate the incidence and slow the progression of disease in elderly individuals. However, modern medicine still has major limitations in the treatment of disease. Many age-related diseases (e.g., type II diabetes) arise from a combination of long-term lifestyle habits and changes in organ metabolic function (12–14). These diseases have a long history of onset, and early symptoms are not significant. However, when diagnosed, there is no cure, which has a significant negative impact on the health of the individual and on the medical burden on society (15). Therefore, improving chronic inflammation through diet is a very important intervention for chronic diseases, in addition to pharmacological treatment.

The gut microbiota is an important target for chronic disease intervention (16, 17). It has been shown that the gut microbiota is involved in the regulation of human phenotypes and health during human evolution (18). For example, *Lactobacillus* can improve autism symptoms in young mice born to obese mothers (19). *Akkermansia* can promote response rates to the tumor microenvironment by modulating STING-type I IFN-dependent monocyte reprogramming (20). Some sources claim that *Akkermansia* in the feces is significantly enriched in patients and mice with type II diabetes (T2DM) after taking metformin (21). Low levels of *Akkermansia* in the intestine may correlate with the thinning of the mucosal layer, which leads to weakened intestinal barrier function (22). The *APOA5* SNP rs651821 in the human genome can lead to an increase in *Bifidobacterium* and can thus modulate metabolic syndrome (23). With advances in high-throughput sequencing technologies, an increasing number of gut microbes are being identified, and their biological functions are becoming better known. As the regulation of health by gut microbes is a slow but important process, targeting specific gut microbes has an important role in the dietary improvement of chronic inflammation (24).

High-amylose starch is a prebiotic that has been widely recognized for its health-improving properties in chronic diseases such as type II diabetes (25, 26). High-amylose starch is mainly derived from the endosperm of plant seeds. The starch produced in the endosperm is synthesized in a special plastid called an amyloplast, unlike the starch produced in other parts of the plant, as those starches are the long-term carbon reservoir for developing seeds (27). Most of the starch consumed by humans comes from the stored starch in the endosperm of cereals (maize, rice, barley, and wheat). Maize (*Zea mays* L.) is the most widely used source of starch due to its genetic diversity (28). Starch is composed of two glucan polymers: amylopectin and amylose. These molecules are generated *via* α-(1 → 4)-bonded glucosyl units that are branched at α-(1 → 6) points. Amylose is a largely linear molecule with only 5–17 α-(1 → 6) branches per molecule (29, 30). Amylopectin comprises the majority of the starch in the granule, ~75%. The complex three-dimensional structure of amylopectin makes it easier to be recognized by amylase in the digestive tract and broken down into smaller molecules (such as maltose). These small molecules are absorbed by the intestine and significantly increase serum blood glucose levels. However, amylose is not easily recognized and degraded by amylase due to its simple three-dimensional structure. In the intestine, amylose is more readily absorbed by microorganisms in the gut, affecting human health by altering the microbial composition (31). Although the substrate for amylose and amylopectin synthesis, namely, ADP-glucose (ADP-Glc), is the same, the synthesis of each polymer requires different enzymes. Amylose is structurally simple and requires only one enzyme for synthesis [granule-bound starch synthase (GBSS)], whereas amylopectin requires the synergistic action of multiple enzymes [AGPase, soluble starch synthase (SS), starch-branching enzyme (SBE), isoamylase (ISA), and pullulanase (PUL)] (32, 33). In maize, *Ae1* and *Sbe1* are two key starch synthesis-related genes that encode the starch-branching enzymes SBEII and SBEI, respectively (34–37). Knockout of either gene alone can restrict normal starch synthesis in the endosperm, resulting in the synthesis of more amylose (38–41). However, while the high-amylose starch synthesis pathway has been well-studied, its functional mechanisms in improving health have not been thoroughly investigated. In addition, since current studies on maize mutants with high-amylose maize starch (HAMS) still focus on mutations in individual genes, which can lead to the inactivation of starch-branching enzymes that are not complete, we wanted to mutate *Ae1* together with *Sbe1* as a way to observe the phenotypic changes.

In the present work, we first created *ae1/sbe1* double mutant maize plants using the CRISPR/Cas9 knockout method. Second, *ae1/sbe1* HAMS was fed to mice on a high-fat diet (HFD), and its phenotype and mechanisms were investigated. We found that high-amylose starch improved gut barrier function by increasing the amount of *Akkermansia* in the colon and reduced chronic inflammation *in vivo*. This resulted in a significant decrease in blood glucose in mice a HFD.

## Materials and methods

### Plant materials

The maize parental lines Hi II and W22 were initially obtained from the Maize Genetics Cooperation Stock Center and were maintained in the Shanghai Key Laboratory of Bio-Energy Crops, Shanghai University. The transgenic lines were generated in this laboratory. All maize plants were cultivated in the experimental field or greenhouse at Shanghai University.

### Construction of the *Ae1/Sbe1*-CRISPR/Cas9 vector

The procedures were adapted according to Qi et al. (42, 43). Two maize U6 promoters and U6 terminators were amplified using specific primers (Supplementary Table 1) and cloned into the *Hind*III site and *Pst*I site of the same pCAMBIA3301 vector with the maize codon-optimized *Cas9* gene. tRNA-gRNA units (TGUs) designed for *Ae1* editing were synthesized by Generey (Generey.com) that containing 4 targets and were cloned into the *BssH*III site between the maize U6 promoter and U6 terminator. Similarly, TGUs for *Sbe1* were cloned into the *Xba*I site between the other U6 promoter and the U6 terminator. This vector includes a *Bar* gene as a selective marker.

### Maize transformation

*Agrobacterium*-mediated maize transformation was carried out according to Frame et al. (44). The positive transgenic lines were acquired after identifying the Bar gene among all regenerated lines using specific primers (Supplementary Table 1). The T1 transgenic progenies were produced by crossing the positive transgenic lines with the inbred line W22. Maize genomic DNA was extracted with the hexadecyltrimethylammonium bromide method from endosperm or leaves. Target regions were amplified with specific primers using Taq 2X Master Mix (Vazyme). Then, the PCR products were cloned into the pGEM-T Easy vector (Promega) for DNA sequencing.

### Extraction of maize protein

Mature kernels were soaked in water, and endosperm was then separated from the embryo and pericarp. Endosperm samples were critically dried to constant weight, powdered in liquid N_2_, and extracted according to the method of Bernard et al. (45). Briefly, a 50-mg sample was incubated overnight in 1 mL of lysis buffer (12.5 mM sodium borate, 1% SDS, 2% b-mercaptoethanol, 1% cocktail, and 1% phenylmethylsulfonyl fluoride) in a 37°C shaker. The mixture was centrifuged for 10 min at 12,000 rpm, after which the supernatant was carefully transferred into a new 1.5-mL centrifuge tube. Proteins were extracted from 50 mg of three pooled endosperm flour samples.

### Immunoblot analysis of maize protein

For the production of the anti-AE1 antibody, 1-375 bp Ae1 was cloned into the pGEX4T-AB1 vector. Similarly, base pairs 1-657 of Sbe1 were cloned into the other pGEX4T-AB1 vector to produce the anti-SBE1 antibody. Antigens for the production of antibodies in rabbits were produced by using the prokaryotic protein expression system of *Escherichia coli* (E. *coli*). All these steps were carried out by Abclonal of China.

Proteins extracted from mutant and wild-type mature kernels were separated by SDS–PAGE. The separated protein samples were then transferred to a polyvinylidene difluoride membrane (0.45 mm; Millipore). The membrane with the protein sample attached was incubated with primary and secondary antibodies. Using the Super Signal West Pico chemiluminescent substrate kit (Pierce), the signal was visualized according to the manufacturer's instructions. The purified AE1, SBE1, and tubulin antibodies were used at 1:1,000, while the secondary antibodies were used at 1:5,000.

### Measurement of amylose

Mature kernels of *ae1/sbe1* and the wild type were soaked in water, and endosperm was then separated from the embryo and pericarp. Endosperm samples were dried to constant weight and pulverized in liquid N_2_, and amylose was extracted and measured using an amyloglucosidase/a-amylase method starch assay kit (Megazyme) according to the instructions and adapted as follows: 1 mL of dimethyl sulfoxide (DMSO) was added to 20 mg of sample and then mixed slowly in a vortex mixer and heated in a boiling bath for 1 min until dispersed to ensure that the starch had no lumps. The tube was heated in boiling water for 15 min and then mixed well-intermittently at high speed. After incubating at room temperature for 5 min, 2 mL of ethanol [95% (v/v)] was added and then mixed. Thereafter, 4 mL of anhydrous ethanol was added and then mixed, and the starch precipitate was formed. The solution was centrifuged at 2,000 × g for 5 min after incubating for 30 min, and the supernatant was discarded. Two milliliters of DMSO was added to the precipitate without any ethanol and then heated in boiling water for 15 min. The solution was transferred to a 25-mL volumetric flask and stabilized with a ConA solution to obtain solution A. ConA (0.5 mL) was added to 1 mL of solution A and then completely mixed. Samples were incubated at room temperature for 1 h and centrifuged at 14,000 × g for 10 min. The supernatant was removed and combined with 3 mL of 100 mM sodium acetate buffer (pH 4.5), which was then mixed and heated in boiling water for 5 min to denature ConA. Then, 0.1 mL of amyltransglucosidase and beta-amylase were added and centrifuged at 2,000 × g for 5 min after 30 min at 40°C. Next, 4 mL of GOPOD reagent was added to 1 mL of supernatant and reacted for 20 min at 40°C. The absorbance of each sample and the D-Glu control at 510 nm was read against the reagent blank. Measurements of all samples were replicated three times.

### High-fat diet feeding

Generally, diabetes can be divided into three types: spontaneous type I diabetes, secondary type II diabetes, and gestational diabetes. Of these, type II diabetes is the most widespread type of disease. The main molecular mechanisms used to establish a mouse model of type II diabetes are the induction of insulin resistance and islet cell dysfunction. The main models are animal models with genetic mutations (*ob/ob* mice) and animal models induced by a high-fat diet. Considering that the population tends to have type II diabetes due to the long-term adoption of a high-fat as well as high-sugar diet, we chose HFD-fed mice as our model.

The animal experiments including HFD-fed mice were approved and authorized by the Shanghai Institute of Family Planning (identification number 2020-34 “Effect of probiotics on body weight and body fat”). The mouse experiment started in 2020. Eight-week-old mice (C57BL/6 mice) were subjected to a constant 12-h day-night cycle and a constant room temperature of 22°C. A total of 30 mice were randomly divided into three groups. Ten mice were fed a normal chow diet (NCD) consisting of 70 kJ% from carbohydrates, 22 kJ% from proteins, and 5 kJ% from fat, and the other twenty mice were fed a HFD consisting of 35 kJ% from carbohydrates, 20 kJ% from proteins, and 45 kJ% from fat. We replaced the 35% carbohydrate corn starch with the high amylose derived from *ae1/sbe1* maize kernels or the normal starch of wild-type maize kernels. These 20 mice were randomly divided into 2 groups. Ten mice from each group were fed different high-fat diets. The mice had excess food and water. The HFD was still maintained during the experimental period. In the oral glucose tolerance test (GTT), we first fasted the mice for 6 h. Adequate drinking water was provided during this period. A 1 g/kg glucose solution was injected into mice by intraperitoneal injection. Blood glucose levels were measured at 0, 15, 30, 60, and 120 min. For organ collection, the mice were sacrificed by cervical dislocation. We used the median with the SEM method for statistical analysis. We used GraphPad for graphing.

### Transmission electron microscopy

The colon tissues were soaked in paraformaldehyde and postfixed in osmium tetroxide. Fixed samples were dehydrated with an ethanol gradient up to 100% and then transferred into a propylene oxide solution and slowly embedded in acrylic resin (London Resin Company). Thin sections (70 nm) were sliced using a diamond knife microtome (Reichert Ultracut E). The sections were placed on 100-mesh copper grids and stained with uranyl acetate for 30 min and with lead citrate for 15 min. The sections were observed with a transmission electron microscope (Hitachi H7600).

### Single-cell RNA sequencing

We randomly selected 3 mouse colon tissues from the HAMS and MS groups for sequencing. During the sample preparation process, we collected ~1 cm of the proximal colon tissues from the HAMS and MS groups, removed the mesentery, and recovered the contents of the colon segment using PBS. The tissue was dissociated into a single-cell suspension by enzyme digestion. Briefly, the tissues were cut into approximately 1 mm^2^ pieces and digested using the Solo^TM^ Tumor Dissociation Kit (JZ-SC-58201) at 37°C for 50 min. After stopping digestion by the addition of excess DMEM, the cell strainer-filtered single-cell solution was kept on ice until it was loaded into a BD Rhapsody cartridge for single-cell transcriptome isolation.

Based on the BD Rhapsody system whole-transcriptome analysis alpha protocol for single-cell whole-transcriptome analysis, microbead-captured single-cell transcriptomes were used to prepare a cDNA library containing cell labels and UMI information. Briefly, double-stranded cDNA was first generated from the microbead-captured single-cell transcriptome in several steps, including reverse transcription, second-strand synthesis, end preparation, adapter ligation, and whole-transcriptome amplification. Then, the final cDNA library was generated from double-stranded full-length cDNA by random priming amplification using a BD Rhapsody cDNA Kit (BD Biosciences, 633773) and the BD Rhapsody Targeted mRNA and AbSeq Amplification Kit (BD Biosciences, 633774). The library was sequenced in PE150 mode (paired-end with 150-bp reads) on an X Ten instrument (Illumina).

Raw reads were processed through the BD Rhapsody Whole-Transcriptome Assay Analysis Pipeline (early access); the processing included filtering by read quality, annotating reads, annotating molecules, determining putative cells, and generating a single-cell expression matrix. Briefly, read pairs with low sequencing quality (too long, too short, low sequencing score, or high single-nucleotide frequency) were first removed at the read quality filtering step. The quality-filtered R1 reads were analyzed to identify the cell label sequence (CL), the molecular identifier sequence (UMI), and the poly-dT tail sequence, and the quality-filtered R2 reads were mapped using STAR (version 2.5.2b) at the read annotation step. Further adjustments were performed using recursive substitution error correction (RSEC) and distribution-based error correction (DBEC) algorithms to remove artifactual molecules arising from amplification bias at the molecule annotation step. Putative cells were distinguished from background noise through a second derivative analysis at the putative cell determination step. Finally, putative cell information was combined with RSEC/DBEC-adjusted molecules to generate a single-cell expression matrix. The pipeline output provided raw gene expression matrices corrected by the RSEC and DBEC algorithms. Among all the matrices, UMI counts per cell corrected by the DBEC algorithm were later used in the clustering analysis.

Raw gene expression matrices from two cartridges were read separately into R (version 3.6.0) and converted to Seurat objects using the Seurat R package (version 3.0.1). CCA integration between two batches was performed with the Seurat R package.

The gene expression matrix was then normalized to the total cellular UMI count. The top 2,000 features were selected as highly variable genes for further clustering analysis. After scaling the data concerning UMI counts, PCA was performed based on the highly variable genes identified in the previous step to reduce dimensionality. In addition, the first 50 principal components were chosen based on the PC heatmap, jackstraw plot, and PC elbow plot to further reduce dimensionality using the UMAP algorithm. Each cluster was then annotated with canonical cluster markers.

Downstream pseudotime trajectory analysis was performed with the Monocle 2 R package.

### RNA extraction and quantitative PCR

An RNA extraction kit (DP430, TIANGEN) was used to extract total RNA from colon tissue, and cDNA was synthesized by a reverse transcription kit (KR123, TIANGEN). The primer pairs for qPCR were designed using BLAST-Primer software (https://www.ncbi.nlm.nih.gov/tools/primer-blast/). The *GAPDH* gene (Ensembl Number: ENSMUSG00000057666) was used as an internal control. For qPCR, the reaction mixture comprised the SYBR Green Mix (208054, QIAGEN), primer mix, and cDNA first-strand template in a final volume of 10 μL. The reactions were performed using a QuantStudio 3 (Thermo Fisher Scientific). The data were analyzed by the ΔΔCt method. We used the mean ± SEM method for statistical analysis and the *t*-test to analyze the significance. We used GraphPad for graphing.

### ELISA

ELISA experiments were mainly performed by referring to the steps in the kit instructions. The kits were purchased from Abcam. Three replicate assays were used for ELISA samples. The average absorbance values for each set of duplicate standards and duplicate samples were calculated. Duplicates should be within 20% of the mean. We used the median±SEM method for statistical analysis and one-way ANOVA with the Bonferroni *post hoc* test to analyze the significance. We used GraphPad for graphing.

### Inflammatory factor detection

The protocol for inflammatory factor detection can be found in the instructions of the mouse Inflammation Antibody Array Kit (Abcam, ab133999). Briefly, the membrane was incubated with blocking buffer. Then, a 1-mL serum sample was added to bind the antibodies that were fixed on the surface of the membrane. Following this step, the membrane was washed with washing buffer five times and then incubated with 1X biotin-conjugated anti-cytokines and 1X HRP-conjugated streptavidin. Finally, the membrane was exposed to X-ray film.

### DNA extraction and PCR amplification

Microbial DNA was extracted from fecal samples using the E.Z.N.A^®^ Fecal DNA Kit (Omega Bio-Tek, Norcross) according to the manufacturer's protocol. The final DNA concentration and purification were determined using a NanoDrop 2000 UV spectrophotometer (Thermo Scientific), and the DNA quality was checked by 1% agarose gel electrophoresis. The V3–V4 hypervariable region of the bacterial 16S rRNA gene was amplified by a thermal cycler PCR system (GeneAmp 9700, ABI). PCRs were performed in triplicate in a 20-μL mixture containing 4 μL of 5 × FastPfu buffer, 2 μL of 2.5 mM dNTPs, 0.8 μL of each primer (5 μM), 0.4 μL of FastPfu Polymerase and 10 ng of template DNA. The PCR products obtained from 2% agarose gels were further purified using the AxyPrep DNA Gel Extraction Kit (Axygen Biosciences).

Purified amplicons were pooled in an equimolar fashion, and paired-end sequencing (2 × 300) was performed on the Illumina MiSeq platform (Illumina) according to the standard protocol of Majorbio Bio-Pharm Technology Co. The experimental results showed that paired-end sequencing was performed on the Illumina MiSeq platform (Illumina) according to the standard protocol.

### Statistical analysis

Statistical analysis was performed using Prism 6 (GraphPad). Data are plotted in the figures as the mean ± SEM. The D'Agostino & Pearson omnibus normality test was used to analyze the normal distribution of the data before the *t*-test. Furthermore, in the process of equal variables analysis, we require *P* > 0.1. This criterion ensures that all data meet statistical requirements. Differences between the two treatment groups were assessed using a two-tailed, unpaired Student's *t*-test. Differences among the three groups [low-density lipoprotein cholesterol (LDL-c), high-density lipoprotein cholesterol (HDL-c), triglyceride, glycosylated serum protein (GSP), insulin, blood glucose, lipopolysaccharide (LPS), alanine transaminase (ALT), aspartate aminotransferase (AST), carbohydrate tolerance test (GTT), lactate dehydrogenase (LDH), and creatine kinase] were assessed using a one-way ANOVA with the Bonferroni multiple comparisons *post hoc* test. A two-way repeated-measures ANOVA with multiple comparisons test was used for the analysis of body weight, glucose level, GTT, and food intake data. Significant differences are indicated in the figures by ^*^p < 0.05. Notable non-significant differences are indicated in the figures by “ns.”

## Results

### Acquisition and analyses of transgenic maize

**Figure 1:**
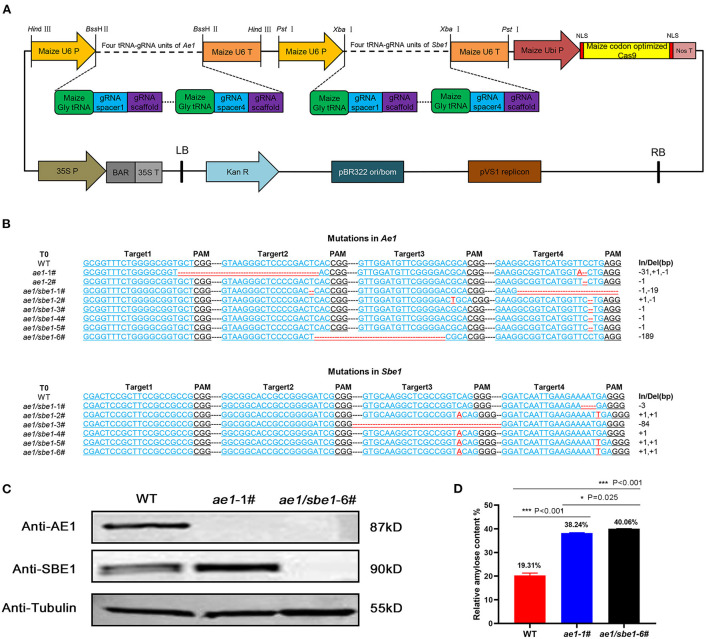
Acquisition and analyses of transgenic maize. **(A)** The *AE1/SBE1*-CRISPR/Cas9 vector based on pCAMBIA3301. P, promoter; T, terminator; Ubi P, ubiquitin promoter; NLS, nuclear localization sequence; Nos T, nopaline synthase terminator; BAR, phosphinothricin R; Kan R, kanamycin resistance gene; LB, left border; RB, right border; **(B)** The editing DNA sequence of transgenic lines. The 20-bp gRNA spacer sequence for the Cas9/gRNA complex is in blue, and the PAM site is in black. Deleted nucleotides and inserted nucleotides are shown in red. The lengths of the insertions or deletions (In/Del) are presented. WT, wild type. **(C)** Immunoblot analysis of AE1 and SBE1 in WT and mutants. Tubulin was employed as an internal control. **(D)** Relative amylose content of WT, *ae1-1#* and *ae1/sbe1*-6# (*n* = 5 biologically independent animals; the data are presented as the mean ± SEM, *P < 0.05, ***P < 0.001, one-way ANOVA with Bonferroni *post hoc* test).

AE1 and SBE1 are two isoenzymes involved in the synthesis of amylopectin in maize (34–37). To obtain a maize line with both *Ae1* and *Sbe1* knocked out, we constructed an *Ae1/Sbe1*-CRISPR/Cas9 vector (Figure 1A) for *Agrobacterium*-mediated maize transformation. The construction of this vector was based on remolded pCAMBIA3301. Eight tRNA-gRNA units (TGUs) of *Ae1* and *Sbe1* are transcribed under the control of U6p. The resulting gRNAs then direct Cas9 to multiple target sites for genome editing (42). After *Agrobacterium*-mediated maize transformation, eight independent T0-positive transgenic lines were acquired after identifying the *Bar* gene among all regenerated lines using specific primers (Supplementary Table 1). To investigate whether the *Ae1* and *Sbe1* genes were edited, their target regions were amplified with specific primers (Supplementary Table 1) flanking the designed target sites. Sequence editing occurred in the target regions of these eight lines (Figure 1B). We identified two *ae1* mutants and six *Ae1/Sbe1* mutants. *ae1*-1# lost 31 bp between target 1 and target 2 in *Ae1* (Figure 1B). *ae1/sbe1-6#* lost 189 bp between target 2 and target 3 in *Ae1* and simultaneously inserted 1 bp at target 3 and target 4 in *Sbe1* (Figure 1B). In transgenic maize, deletion of large segments and point mutations often cause abnormal protein translation and affect the function of the protein. Therefore, *ae1*-1# and *ae1/sbe1*-6# were chosen for the subsequent studies.

To verify the presence of the AE1 and SBE1 proteins in *ae1* and *ae1/sbe1* mutants, western blot analysis was carried out (Figure 1C). The AE1 protein was absent in the *ae1* mutant, and neither AE1 nor SBE1 protein could be detected in the total grain protein of the *ae1/sbe1* mutant. To explore whether the starch composition was changed in the mutants, an amylose assay experiment was performed. We chose *ae1/sbe1*-6# to perform the amylose assay, as its two proteins involved in the synthesis of amylopectin were both absent. The results showed that compared with WT, the amylose content of *ae1/sbe1*-6# mutant was increased by ~2-fold (Figure 1D). Furthermore, we also found that the *ae1* mutants showed a significant increase in the seed HAMS content (38%), but the *ae1/sbe1* kernels demonstrated an even greater increase in the HAMS content of 40%. *ae1* and *ae1/sbe1* mutations also differed significantly from each other (*P* = 0.025). Overall, we obtained transgenic maize, *ae1/sbe1*-6#, with a higher amylose content using the CRISPR/Cas9 editing system.

### *Ae1/Sbe1* maize-derived high-amylose starch may alleviate symptoms of hyperglycemia caused by a HFD

**Figure 2:**
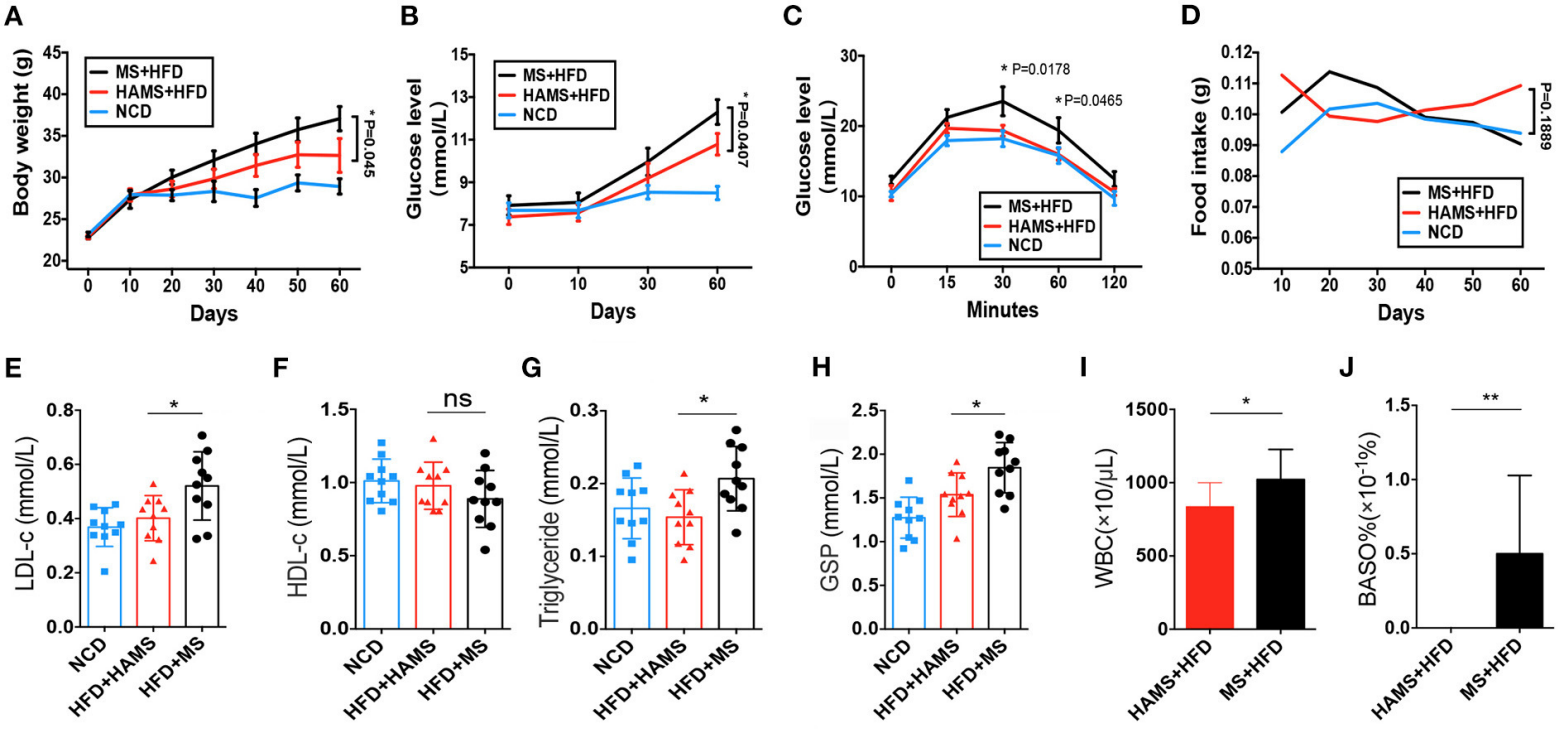
HAMS ameliorates HFD-induced hyperglycemia. **(A)** Body weight data from NCD, HAMS, and MS mice (*n* = 10 biologically independent animals per group, the data are presented as the median ± SEM, *P < 0.05). Since each group had data at 4 different time points, we used two-way ANOVA with the Bonferroni *post hoc* test method to compare the HAMS group and the MS group. **(B)** Blood glucose levels in HAMS and MS mice after HFD and NCD (*n* = 10 biologically independent animals per group, the data are presented as the median ± SEM, *P < 0.05). We used two-way ANOVA with the Bonferroni *post hoc* test method to compare the HAMS group and the MS group. **(C)** Blood glucose levels in HAMS and MS mice after glucose injection (*n* = 10 biologically independent animals per group, the data are presented as the median ± SEM, *P < 0.05). We used two-way ANOVA with the Bonferroni *post hoc* test method to compare the HAMS group and the MS group. **(D)** Food intake in HAMS and MS mice after HFD and NCD (*n* = 10 biologically independent animals per group, the data are presented as the median ± SEM, *P < 0.05). We used two-way ANOVA with the Bonferroni *post hoc* test method to compare the HAMS group and the MS group. **(E–H)** Serum cholesterol-related indicators (*n* = 10 biologically independent animals per group, the data are presented as the median ± SEM, *P < 0.05, one-way ANOVA with Bonferroni *post hoc* test. *N* = 10 NCD animals are also shown). **(I,F)** Changes in WBC and BASO% in the HAMS and MS groups (*n* = 5 biologically independent BD5115 animals vs. *n* = 5 SM animals; the data are presented as the median ± SEM, *P ≤ 0.05, Student's *t*-test). ns, not significant.

HAMS is generally considered to improve the symptoms of hyperglycemia caused by a HFD. However, the molecular mechanisms are not fully understood. Here, we tailored a high-fat diet (45% fat) with *ae1/sbe1* maize-derived HAMS and wild-type maize starch (MS) and fed the diets to wild-type C57BL/6J mice. Body weight, blood glucose, and food intake were continuously monitored over the course of the experiment, which lasted 60 days. A glucose tolerance test was also performed in the last week. The weight data showed that the HFD-fed mice weighed significantly more than the mice on the normal chow diet (NCD) (Figure 2A). The mice in the HAMS group weighed less than the mice in the MS group. For the blood glucose index, we found that the blood glucose of the mice on the HFD was significantly greater than that of the mice on the NCD (Figure 2B). In contrast, the mice in the HAMS group had lower blood glucose levels than those in the MS group. In the glucose tolerance experiment, we administered glucose at a concentration of 1 g/kg intraperitoneally and measured the blood glucose of the mice. The blood glucose of the mice in the MS group showed the highest peak at 30 min after intraperitoneal injection (Figure 2C). The blood glucose of the mice in the HAMS and NCD groups was significantly lower than that of the MS group at both 30 and 60 min. As the decrease in blood glucose was associated with high-fat dietary intake, to exclude the effect of food intake on the blood glucose of the mice, we also measured the food intake of the mice in the 3 groups continuously. The results showed that food intake remained relatively stable in all 3 groups of mice over 60 consecutive days of the experiment and was not significantly different (Figure 2D). Therefore, we ruled out the possibility that the differences in the experimental data were due to differences in food intake.

After dissection of the mice, we also measured parameters related to lipids and blood glucose in the serum of the mice. We found that low-density lipoprotein (LDL), as well as triglycerides, were significantly decreased in the HAMS group compared to the MS group (Figures 2E,G). However, there was no significant difference in high-density lipoprotein (HDL) levels (Figure 2F). Glycosylated hemoglobin was significantly reduced in the HAMS group (Figure 2H). The serum results validated the ameliorative effect of HAMS on blood glucose in HFD-fed mice. Finally, some studies have reported that white blood cells (WBCs) and basophils are altered in hyperglycemia (46), so we also assessed WBCs and basophils in mice. The results showed that peripheral blood leukocytes, as well as basophils, were differentially decreased in the HAMS group (Figures 2I,J). This result is similar to that already reported and further confirms the ameliorative effect of high-amylose starch on hyperglycemia.

### HAMS promotes the proliferation of intestinal epithelial cells and reduces the number of immune cells in the gut

**Figure 3:**
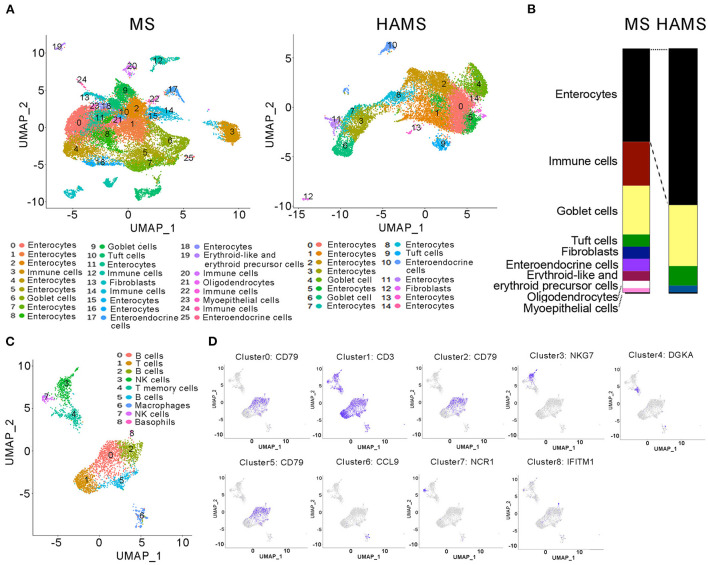
Single-cell RNA sequencing of colon tissues. **(A)** UMAP analyses of colon tissues (*n* = 3 biologically independent HAMS animals vs. *n* = 3 MS animals) from the HAMS group and MS group are shown. Each color represents cells with a different subidentity. **(B)** The proportions of different cell types in the colon tissues of mice in the HAMS group and MS group. **(C)** UMAP analyses of immune cells in the MS group. Each color represents cells with a different subidentity. **(D)** UMAP analyses of marker genes in the MS group.

In general, food entering the intestine is first absorbed by intestinal epithelial cells (IECs) before it enters the systemic circulation and affects systemic health. Therefore, studies of the function of intestinal tissues can often explain the specific reasons why food regulates systemic health. Studying intestinal tissues at the cell biology level as well as the molecular biology level helps us to explore the molecular mechanisms by which high-amylose starch improves blood glucose. Therefore, after dissecting mice in the HAMS group and the MS group, we obtained mouse colon tissue from the mid-section and sequenced the tissue for single-cell RNA. After cell sorting, 26 cell subtypes were identified in the MS group, including IECs, immune cells, goblet cells, tuft cells, and intestinal endocrine cells. In the HAMS group, 15 cell subtypes were identified, including IECs, goblet cells, tuft cells, and intestinal endocrine cells (Figure 3A). In the HAMS group, no immune cells were identified, and the proportion of IECs was greater than that in the MS group (Figure 3B). This finding implies that colonic tissue in the HAMS group may have a more intact gut barrier function compared to the MS group. After reclustering the immune cells in the MS group with the UMAP method, we identified nine different immune cells, including B cells, T cells, NK cells, macrophages, and neutrophils (Figures 3C,D). A HFD may impair the integrity of IECs, thereby compromising gut barrier function. We hypothesized that HAMS could promote the proliferation of intestinal epithelial cells to improve the gut barrier. This improvement in gut barrier function significantly suppressed inflammation and reduced the number of immune cells in the gut.

### *Akkermansia* is significantly enriched in the HAMS group

**Figure 4:**
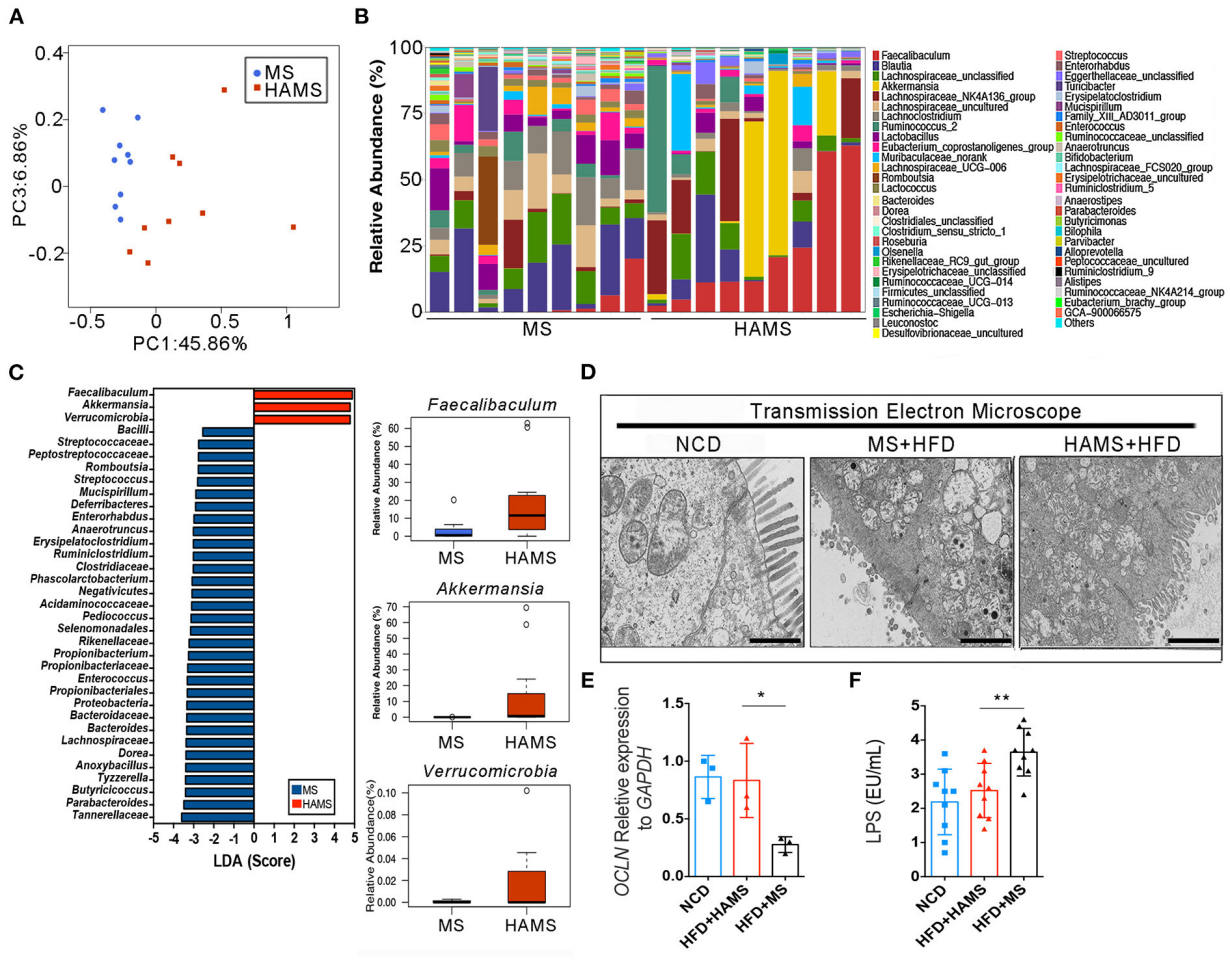
*Akkermansia* was significantly enriched in the HAMS group. **(A)** PCoA of the gut microbiota (*n* = 9 biologically independent HAMS animals vs. *n* = 9 MS animals) from the HAMS group and MS group are shown. **(B)** Relative abundances of the gut microbiota (*n* = 9 biologically independent HAMS animals vs. *n* = 9 MS animals) from the HAMS group and MS group are shown. **(C)** *Faecalibaculum, Akkermansia*, and *Verrucomicrobia* were enriched in the HAMS group. **(D)** Representative electron microscopy images of freshly collected colon tissues from HAMS and MS mice. The bar represents 20 μm. **(E)** Changes in the expression of *OCLN* were detected by qPCR (*n* = 3 biologically independent HAMS animals vs. *n* = 3 MS animals; the data are presented as the mean ± SEM, *P ≤ 0.05, Student's *t*-test). **(F)** Serum LPS of the three groups of mice (*n* = 9 biologically independent HAMS animals vs. *n* = 9 MS animals; the data are presented as the median ± SEM, **P < 0.01, one-way ANOVA with Bonferroni *post hoc* test. *N* = 9 NCD animals are also shown).

The gut microbiota is an important regulatory element in intestinal health. The effect of modulating the gut microbiota on the improvement in the gut barrier has been reported in many previous works (47). To determine the regulatory role of HAMS on IECs, we first sequenced 16S rRNA in mouse feces from the HAMS and MS groups. PCoA completely separated the HAMS and MS groups, implying a large difference in the microbiota contents between the HAMS and MS groups (Figure 4A). When analyzing the microbiota contents of separate samples, we also found that the HAMS group differed more in microbiota diversity as well as in the abundance of some microorganisms (Figure 4B). In analyzing the two groups of differential strains using LDA, we identified three microorganisms, namely, *Faecalibaculum, Akkermansia*, and *Verrucomicrobia*, as being significantly enriched in the HAMS group (Figure 4C). *Akkermansia* has been widely implicated in previous work as being involved in gut barrier repair, human immune regulation, and other functions (48). This outcome contributes to an improved inflammatory response, thereby suppressing hyperglycemia. To test the hypothesis above, we first used transmission electron microscopy (TEM) to observe the changes in colonic tissue. In the NCD group, we found that the IECs were intact and well-structured (Figure 4D). In contrast, in the MS group, the IECs were severely damaged, and no intact IEC structure could be seen. Although the IECs were also partially damaged, the structure was relatively clear in the HAMS group. In addition, we also analyzed the expression of *Occludin*, a key gene of the gut barrier, by qPCR. The results showed that the expression of the *OCLN* gene was significantly higher in the HAMS group (Figure 4E). Plasma lipopolysaccharide (LPS) is an indirect indicator to assess the permeability of the gut barrier (49). We also performed a quantitative analysis of LPS (Figure 4F). The results confirmed a significant decrease in LPS levels in the HAMS group compared to the MS group. All these results imply that HAMS can improve the structure and function of the gut barrier.

### HAMS improves gut barrier function and reduces the systemic inflammatory response

**Figure 5:**
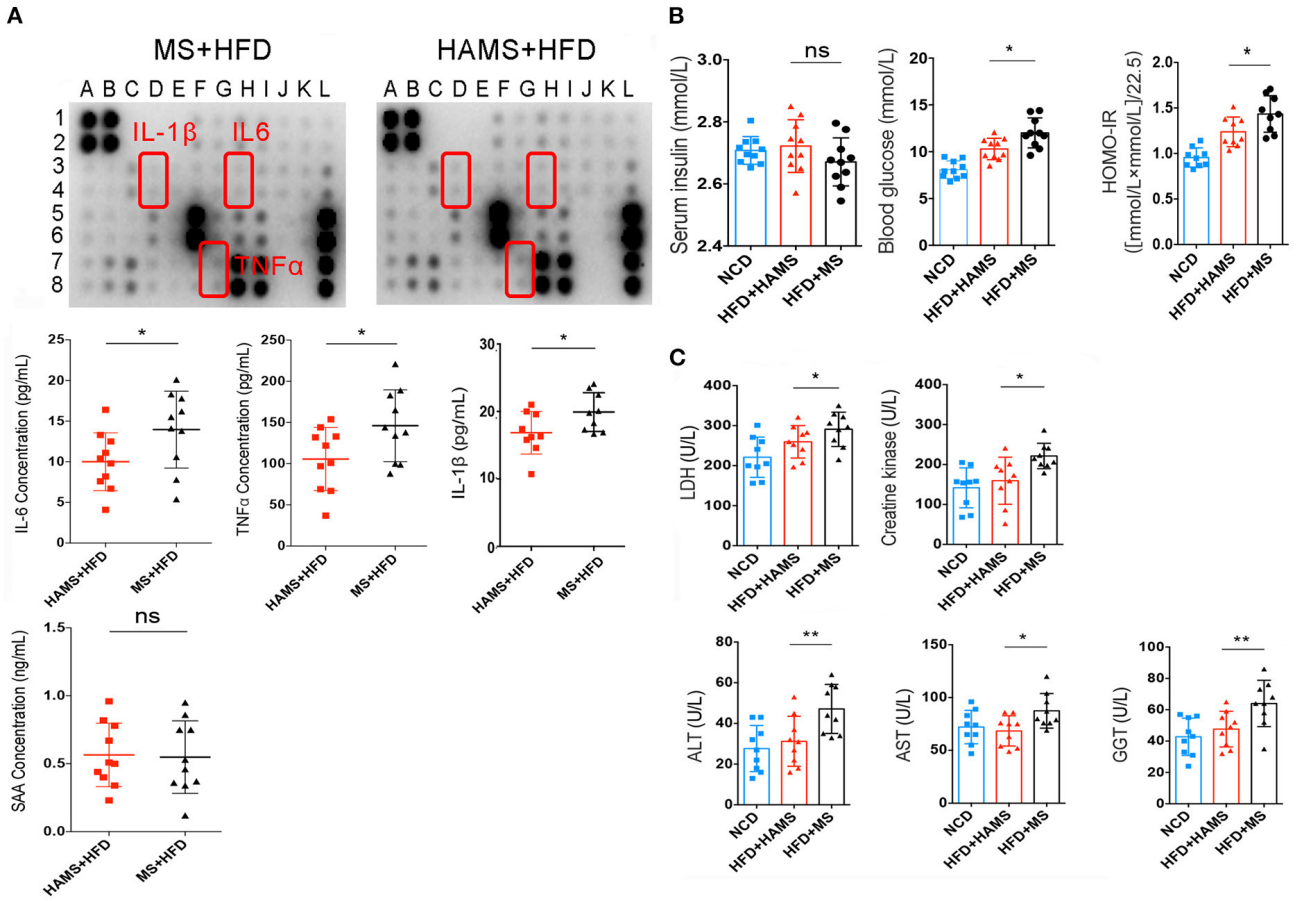
HAMS improves gut barrier function and reduces the systemic inflammatory response. **(A)** Inflammatory factor antibody array and ELISAs were both used to detect the expression of inflammatory factors in serum (*n* = 10 biologically independent animals per group, the data are presented as the median ± SEM, *P < 0.05, Student's *t*-test. *N* = 10 for NCD are also shown). Detailed information on the inflammatory factors can be seen in Supplementary Table 1. **(B)** Serum insulin, blood glucose, and HOMA-IR were all analyzed (*n* = 10 biologically independent HAMS animals vs. *n* = 10 MS animals; the data are presented as the median ± SEM, *P ≤ 0.05, one-way ANOVA with Bonferroni *post hoc* test, *n* = 10 for NCD are also shown). **(C)** Organ and tissue damage indicators were all analyzed in the NCD, HAMS, and MS groups (*n* = 9 biologically independent animals per group, the data are presented as the median ± SEM, *P < 0.05, **P < 0.01, one-way ANOVA with Bonferroni *post hoc* test. *N* = 9 for NCD are also shown).

To further confirm the systemic modulation of the improved gut barrier in the HAMS group, a semiquantitative analysis of serum from the HAMS and MS groups was performed using an inflammatory factor antibody array. The antibody array allowed the analysis of 40 inflammatory factors, including IL-6, IL-1β, and TNFα. Most likely because the differences in inflammatory factor expression were not particularly large in serum, the results of the antibody array did not identify significant differences (Figure 5A). We then performed a quantitative analysis of some classical inflammatory factors using ELISA. The results showed that the expression of IL-6, TNFα, and IL-1β was significantly decreased in the HAMS group (Figure 5A). In addition, serum amyloid A (SAA) is an indicator of acute infection *in vivo*. Elevated levels of this protein indicate acute inflammation in the body. In our tests for SAA, we did not find acute inflammation in the mice. These data suggest that HAMS may improve gut barrier function by increasing the amount of *Akkermansia* in the intestine and alleviating the inflammatory response *in vivo*.

It has also been reported that improving the gut barrier function may also modulate insulin tolerance (50). Therefore, we continued to measure serum insulin and serum glucose. The results showed a significant decrease in serum glucose and the insulin resistance index in the HAMS group compared to the MS group (Figure 5B). In contrast, there was no significant change in serum insulin in the HAMS group. The regulation of blood glucose is associated with serum insulin concentrations, serum insulin antibodies, and insulin receptors. Serum insulin concentrations were unchanged and blood glucose was decreased in the HAMS group, suggesting that HAMS could increase the sensitivity to insulin by improving the gut barrier.

To confirm this possibility, serum lactate dehydrogenase (LDH) and creatine kinase were quantified in serum. LDH and creatine kinase are markers of tissue damage and muscle-specific damage, respectively (51, 52). In contrast, muscle converts large amounts of serum glucose into glycogen and is an important way of storing glucose in the blood. The sensitivity of muscle to insulin determines the efficiency with which blood glucose is converted to glycogen. Here, we assayed the activity of these two enzymes to determine the change in the status of muscle tissue following HAMS intake (Figure 5C). We found that, similar to liver enzymes, both LDH and creatine kinase were significantly elevated in the MS group and that HAMS reduced the levels of these enzymes in serum, thereby reducing muscle damage and increasing the sensitivity to insulin.

Gamma-glutamyl transferase (GGT), aspartate aminotransferase (AST), and alanine aminotransferase (ALT) are all hepatic enzymes whose elevated activity is associated with adverse changes in glucose and lipid metabolism; therefore, these enzymes are considered to be markers of inflammation and risk factors for insulin resistance and the development of T2DM (53, 54). We assessed the trends in these markers following HAMS intake (Figure 5C). Our results revealed that GGT, AST and ALT were significantly elevated in the MS group and that HAMS reduced the levels of these enzymes in the serum, thereby reducing the inflammatory response *in vivo*.

## Discussion

HAMS has been thought to be involved as a prebiotic in improving the development of type II diabetes for many years (25, 26). However, to date, although stably inherited *ae1* and *sbe1* mutants have been obtained from rice, wheat, maize, and pea, only maize-derived RSII high-amylose starch has been widely licensed for food applications (40, 55, 56). In the present work, we targeted the prebiotic function of HAMS in depth. For the first time, we conducted phenotypic studies on *ae1/sbe1* double mutant HAMS in plant physiology and biochemistry as well as functional studies on health regulation. Our serum data not only confirmed the ameliorative effect of HAMS on hyperglycemia, but our quantitative analysis of inflammatory factors also confirmed the strong ameliorative effect of HAMS on chronic inflammation. Considering that chronic inflammation is a widespread subhealth condition in the human body and a potential pathogenic risk factor for a variety of diseases, we suggest that HAMS also has a beneficial effect on chronic inflammation. We believe that HAMS also has the potential to prevent and improve other diseases caused by chronic inflammation. This notion needs to be further investigated in other animal models.

Although HAMS has previously been identified as having prebiotic health functions, its molecular mechanisms are not fully understood. Earlier theories suggested that HAMS could produce short-chain fatty acids through digestion by the gut microbiota in the large intestine, thereby increasing insulin sensitivity (57, 58). However, these studies are more clinical in nature and do not fully explain the functional mechanisms of HAMS. In this work, our TEM results first confirmed the relationship between HAMS and IECs. The HFD significantly disrupted the structure of the intestinal barrier, leading to a significant structural deficit in IECs. This phenotype could be partially improved by HAMS. Although the IEC structure in the HAMS group did not return to the full morphology of the NCD group in our TEM results, there was still a very significant recovery compared to that of the MS group. In addition, we also used single-cell RNA sequencing to investigate the mechanisms of the healthy function of HAMS. Considering that there are a large number of different cells in colon tissue, such as IECs, immune cells, intestinal endocrine cells, and goblet cells, single-cell RNA sequencing can better classify and count the different types of cells. This technique helped us to observe the effect of HAMS on intestinal cell composition. The single-cell RNA sequencing results confirmed that the content of IECs was significantly higher in the HAMS group, and the content of immune cells (including B cells, T cells, NK cells, and macrophages) was significantly lower. Combined with the data from 16S rRNA sequencing as well as TEM, we concluded that the HFD led to a compromised intestinal barrier and increased chronic inflammation *in vivo* and that HAMS in the colon promoted the proliferation of *Akkermansia*, which in turn reduced chronic inflammation *in vivo* by repairing the compromised intestinal barrier function and increased muscle sensitivity to insulin. This is a systematic finding. We have integrated aspects of the gut microbiota and insulin sensitivity from earlier studies with the gut barrier and chronic inflammation. This research could explain in detail the functional mechanisms of HAMS in improving hyperglycemia. Furthermore, our study did not find an effect of HAMS on food intake in mice. This refutes the belief that HAMS functions to lower blood glucose by increasing satiety. Overall, our mechanistic study could be of great assistance to the future industrialization of HAMS as a prebiotic. In addition, we also found that *Faecalibaculum* was significantly enriched with *Verrucomicrobia* in the HAMS group. *Verrucomicrobia* is widely believed to be present in soil and water, and its health functions in humans need to be further confirmed (59–61). In contrast, some strains of *Faecalibaculum* (*Faecalibaculum rodentium*) were previously reported to inhibit the growth of intestinal tumors through the release of SCFAs (62). This finding implies that HAMS may also have a potential positive role in inhibiting colonic adenomas.

In addition, we used plant genetics, medicine, and bioinformatics assays to investigate the health functions of HAMS in this work. This is a typical interdisciplinary research result in the life sciences. In the field of nutritional health, considering that many prebiotics are of plant origin, it is necessary to start targeted creation at the plant breeding level and to systematically study prebiotics using biomedical, microbiological, food science, and bioinformatics tools. This approach will help us to achieve efficient development and industrialization of prebiotics.

## Data availability statement

The datasets presented in this study can be found in online repositories. The names of the repository/repositories and accession number(s) can be found in the article/Supplementary material.

## Ethics statement

The animal study was reviewed and approved by the Shanghai Laboratory Animal Management Office [SYXK (Shanghai) 2017-0008].

## Author contributions

WQ, RS, and ZQ: conceived and designed the experiments. WQ, JL, TY, and SH: performed the experiments. WQ and JL: analyzed the data. RS: seed provision. WQ, JL, and ZQ: wrote the paper. All authors contributed to the article and approved the submitted version.

## Accession numbers

The gene expression data obtained by using next-generation RNA sequencing are deposited into the SRA database under accession number PRJNA846362.

## Funding

This work was supported by the Shanghai Rising-Star Program (Grant 18QB1400100 to ZQ) and the National Key Research and Development Program of China (Grant 2016YFD0100503 to WQ).

## Conflict of interest

Author ZQ is employed by Bright Dairy & Food Co., Ltd. The remaining authors declare that the research was conducted in the absence of any commercial or financial relationships that could be construed as a potential conflict of interest.

## Publisher's note

All claims expressed in this article are solely those of the authors and do not necessarily represent those of their affiliated organizations, or those of the publisher, the editors and the reviewers. Any product that may be evaluated in this article, or claim that may be made by its manufacturer, is not guaranteed or endorsed by the publisher.
