# Supplementary material for: Ae1/Sbe1 maize-derived high amylose improves gut barrier function and ameliorates type II diabetes in high-fat diet-fed mice by increasing Akkermansia
Source: Front Nutr. 2022 Sep 29;9:999020. doi: 10.3389/fnut.2022.999020 (PMC9556726; doi:10.3389/fnut.2022.999020)
Supplement: Supplementary file 1 [file Table_1.docx]

**Table S1. Primers for this study**

| **Purpose** | **Name** | **Sequences(5’-3’)** |
| --- | --- | --- |
| Vector construction | U6P-F1 | AAGCTTCAGCAAATAATGGCATCCGA |
|  | U6P-R | GCGCGCGATGCGGTGCTTCCGGTTTG |
|  | U6P-F2 | CTGCAGCAAATA ATGGCATCCGATG |
|  | U6T-F1 | GCGCGCAATTTTTTTGCGGATTTGCG |
|  | U6T-R1 | AAGCTTGGGCTAAAGGAAAAAAATGT |
|  | U6T-F2 | TCTAGAAATTTTTTTGCGGATTTGCG |
|  | U6T-R2 | CTGCAGGGGCTAAAGGAAAAAAATGT |
| Identification of Bar gene | Bar-F | GAAGTCCAGCTGCCAGAAAC |
|  | Bar-R | GCACCATCGTCAACCACTAC |
| Identification of target regions | Ae1-cas9-F | CGCTGGGGTTTTAGCATTGG |
|  | Ae1-cas9-R | ACGAACCTCCAGTTCATCCG |
|  | Sbe1-cas9-F | TCCGGCACCCGATATAAAGC |
|  | Sbe1-cas9-R | GGCATGGGCAGGTGCTATAA |
