# Supplementary figures and images for: Ae1/Sbe1 maize-derived high amylose improves gut barrier function and ameliorates type II diabetes in high-fat diet-fed mice by increasing Akkermansia
Source: Front Nutr. 2022 Sep 29;9:999020. doi: 10.3389/fnut.2022.999020 (PMC9556726; doi:10.3389/fnut.2022.999020)

**Table S2.** **The detailed information on inflammatory factors.**


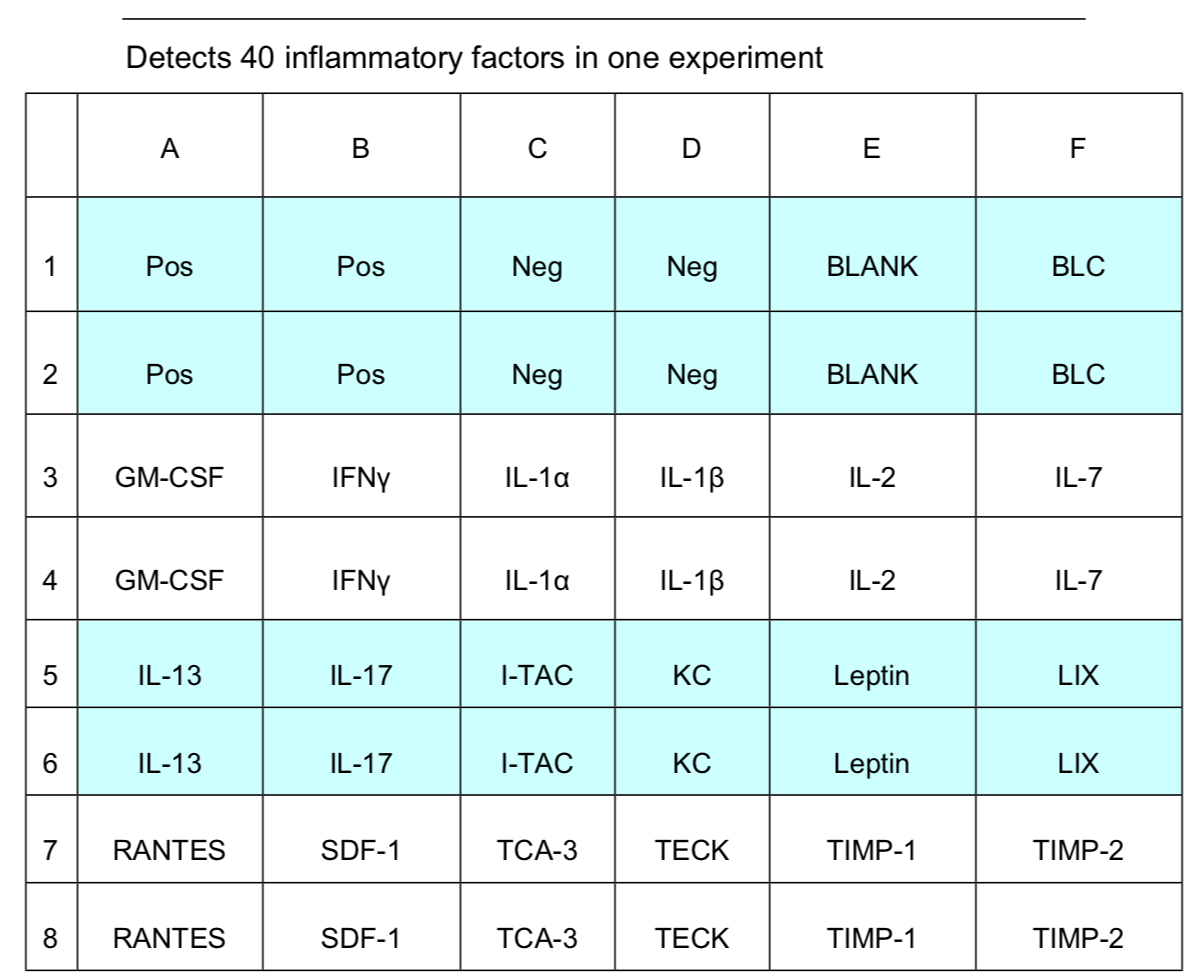


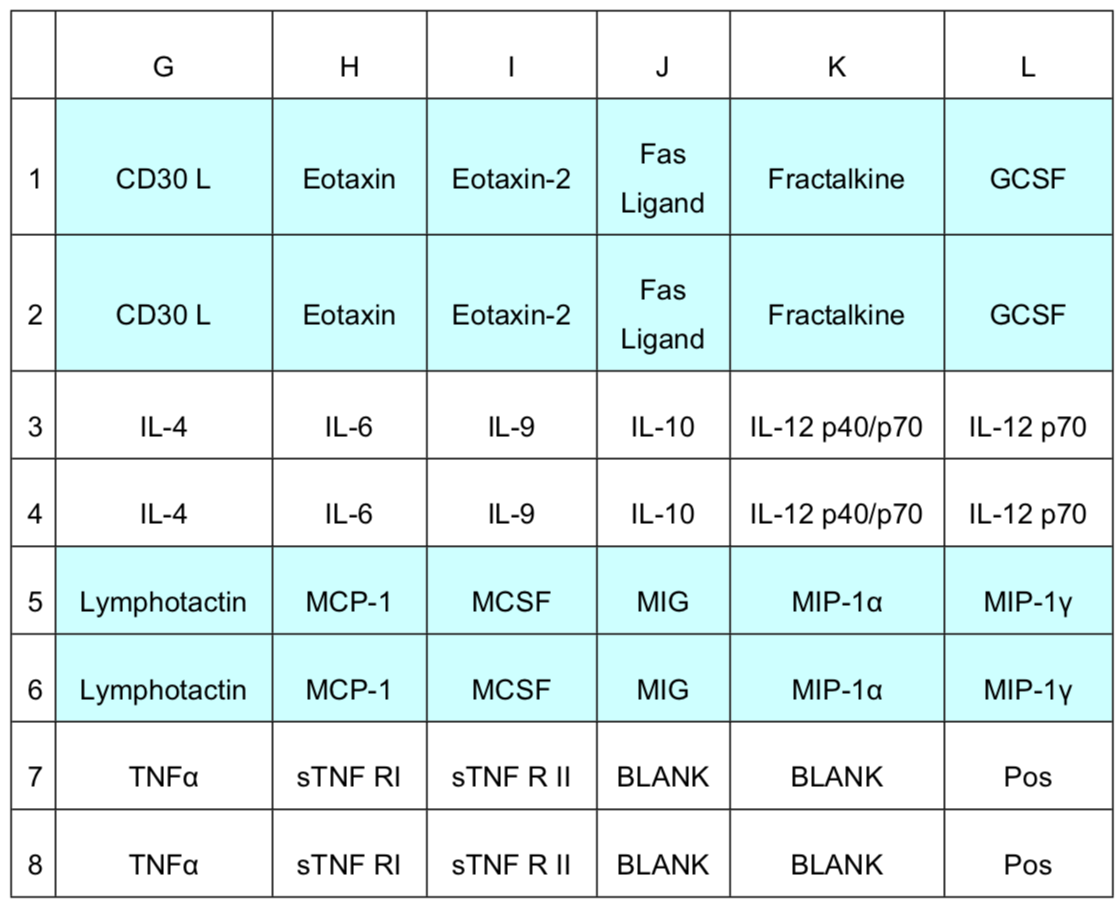

Supplement: Supplementary file 2 [file Table_2.docx]
